# Supplementary material for: Temporal development of Drosophila embryos is highly robust across a wide temperature range
Source: J R Soc Interface. 2018 Jul 11;15(144):20180304. doi: 10.1098/rsif.2018.0304 (PMC6073635; doi:10.1098/rsif.2018.0304)
Supplement: Figure supplement [file rsif20180304supp1.pdf]

Supplementary material:

Temporal development of *Drosophila* embryos is highly robust across a wide temperature range

Jeronica Chong[1,*], Christopher Amourda[1,*] and Timothy E. Saunders[1,2,3,#]

[1] Mechanobiology Institute, National University of Singapore, Singapore
[2] Department of Biological Sciences, National University of Singapore, Singapore
[3] Institute of Molecular and Cell Biology, A*Star, Proteos, Singapore
[*] These authors contributed equally to this paper
[#] Correspondence: dbsste@nus.edu.sg

Supplementary figures

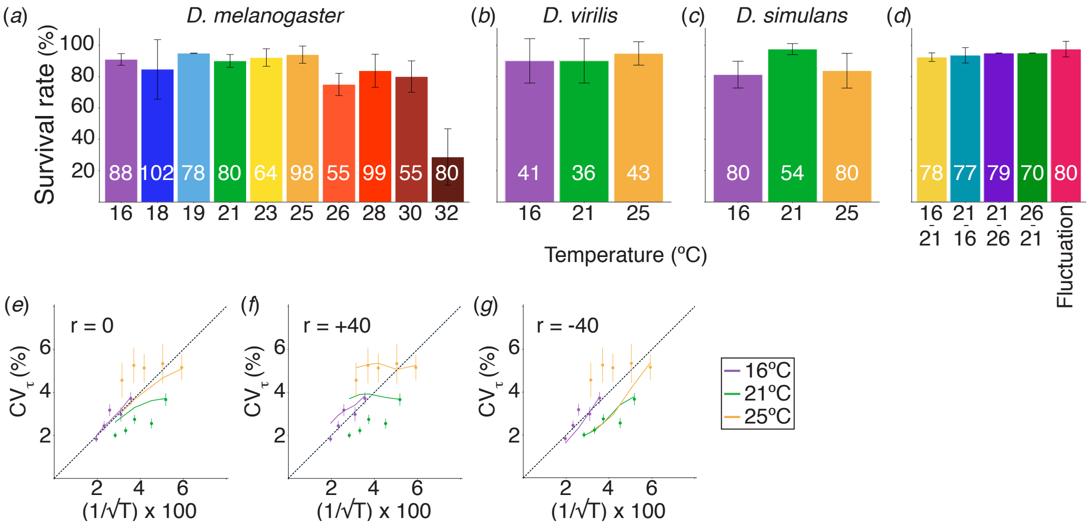

Figure S1: Experimental setup and controls

(a-d) Survival rate of *D. melanogaster (a), D. virilis (b), D. simulans (c)* and *D. melanogaster* with temperature perturbations *(d)*. Numbers embedded in bars correspond to number of embryos. *(e)* Simulations of developmental trajectories in *D. melanogaster* without history dependence at any temperature. *(f)* Simulations of developmental trajectories in *D. melanogaster* with reinforcement of the history dependence at all temperatures. *(g)* Simulations of developmental trajectories in *D. melanogaster* with negative feedback in the history dependence at all temperatures. Errors on CV_τ are s.d., calculated using Bootstrapping as described in Methods.

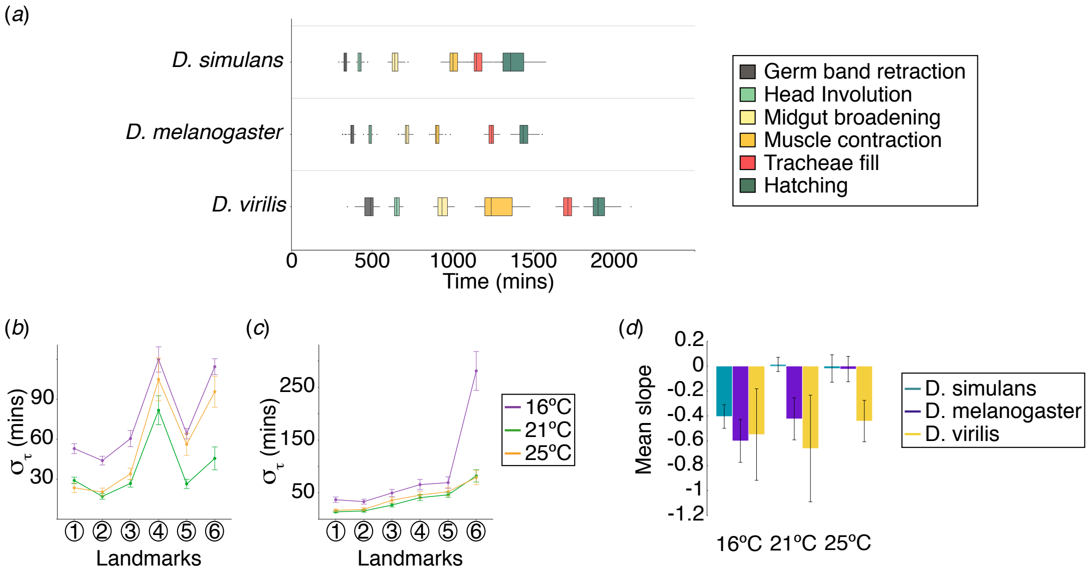

Figure S2: Temporal development of *Drosophila* species at 16ºC, 21ºC and 25ºC *(a)* Distribution of developmental times at each landmark scored for *D. simulans*, *D. melanogaster* and *D. virilis* at 25ºC. *(b,c)* Absolute error in developmental time at each landmark for *D. virilis (b)* and *D. simulans (c)* at 16, 21 and 25ºC. (*d*) Fitting $CV_\tau = a\tau^s$ for each species at each temperature. Mean slope corresponds to value of s for fitting between germband retraction to trachea filling. All error bars are s.d.

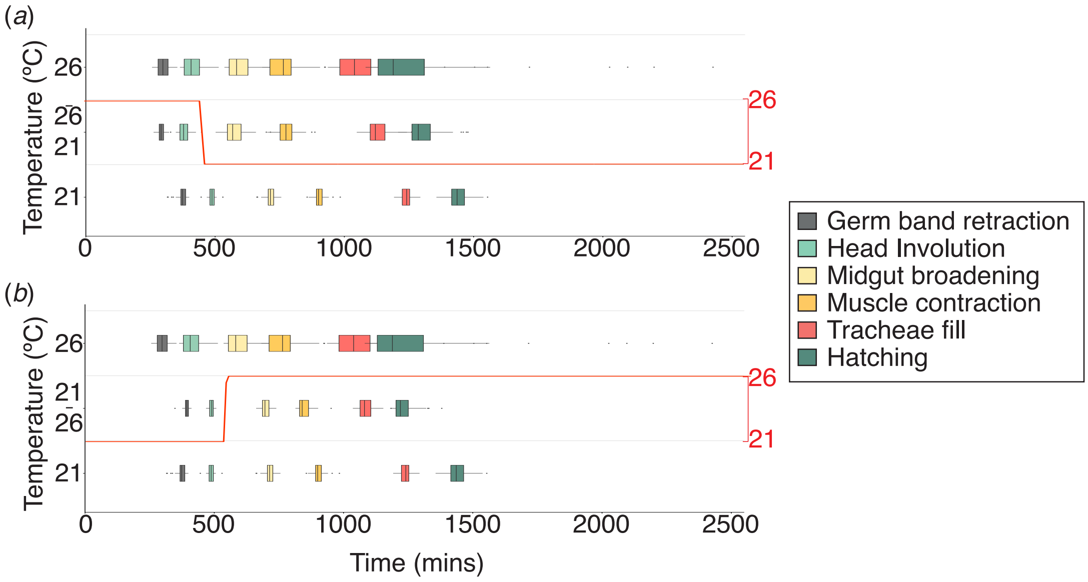

Figure S3: Further analysis of temperature shift

(a,b) Distribution of developmental times at each landmark scored for *D. melanogaster* under 21 to/from 26ºC temperature shift. Temperature was shifted after head involution and is represented by a red curve.

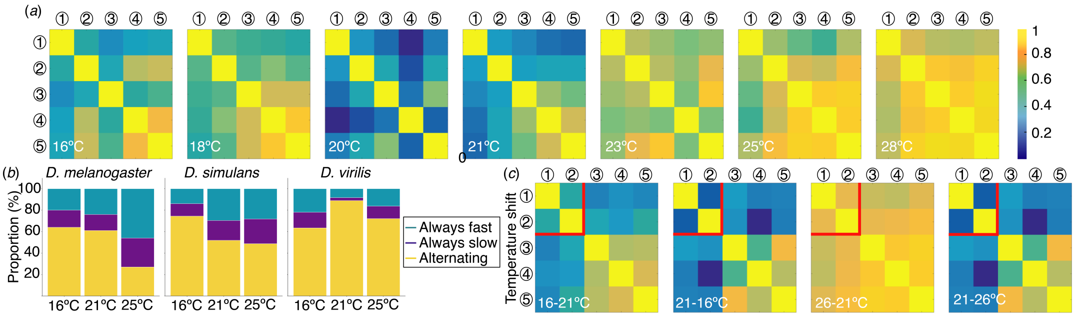

Figure S4: History dependence in *D. melanogaster*

(a) Covariance matrices for all temperatures recorded in *D. melanogaster*. *(b)* Proportion of embryos always faster than the mean (turquoise), slower than the mean (purple) or alternating between being faster or slower (yellow) when excluding germband retraction from analysis. *(c)* Covariance matrices for temperature shift experiments recorded in *D. melanogaster*.

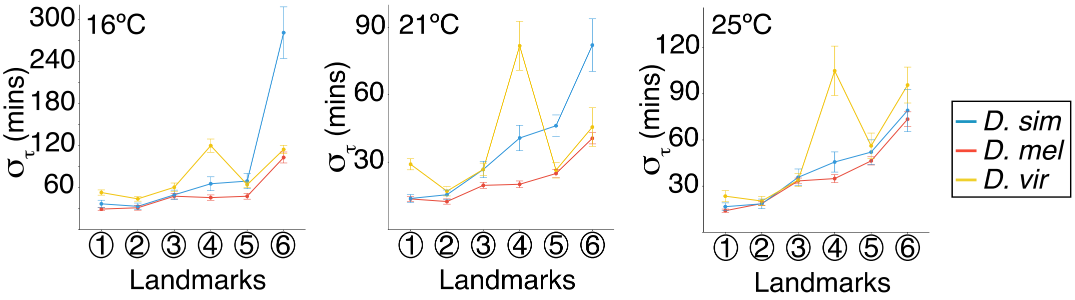

Figure S5: Absolute standard error of the different species

Absolute error in developmental time at each landmark for *D. melanogaster, D. virilis* and *D. simulans* at 16, 21 and 25ºC. Errors are s.d.

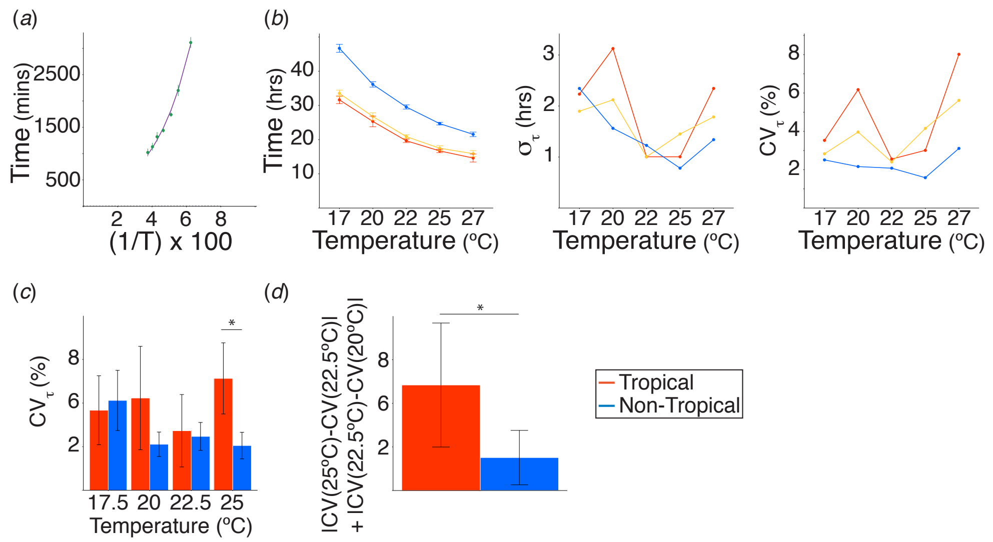

Figure S6: Comparison to previous measurements of temporal trajectories
(a) Time to hatching from cephalic furrow formation for *D. melanogaster* at different temperatures. Purple curve is a fit to the Arhennius equation, $\tau = \tau_0 e^{T_0/T}$. *(b)* All data extracted from figure 5 of Kuntz and Eisen, PLoS Genetics 2014. Left: Measured time to tracheae filling at different temperatures in *D. simulans* (red), *D. melanogaster* (yellow) and *D. virilis* (blue). Centre: Measured temporal error, and right: coefficient of variation. Error bars are standard deviation. *(c)* CV_τ for the variability in times to trachea filling from the data set in Kuntz and Eisen, PLoS Genetics 2014. Data is separated into tropical (red, n=6) and non-tropical (blue, n=4) species, as described in main text. Error bars are s.d.. *(d)* Comparing the variability in CV_τ from 20ºC to 25ºC between tropical and non-tropical species. Non-tropical species (blue) have small change in CV_τ (<2% on average) across 20ºC to 25ºC, whereas tropical species (red) display larger variability in the recorded CV_τ with temperature. Error bars s.d., and significance determined by t-test comparison of the means.
